# Unilateral Maximal Isometric Hex Bar Pull Test: Within-Session Reliability and Lower Body Force Production in Male and Female Freeski Athletes

**DOI:** 10.3389/fspor.2021.715833

**Published:** 2021-08-09

**Authors:** Jonathan McPhail, Basílio A. M. Gonçalves, Jörg Spörri, Vesa Linnamo

**Affiliations:** ^1^Faculty of Sport and Health Sciences, University of Jyväskylä, Jyväskylä, Finland; ^2^School of Allied Health Sciences, Griffith University, Brisbane, QLD, Australia; ^3^Sports Medical Research Group, Department of Orthopaedics, Balgrist University Hospital, University of Zurich, Zurich, Switzerland; ^4^University Centre for Prevention and Sports Medicine, Department of Orthopaedics, Balgrist University Hospital, University of Zurich, Zurich, Switzerland

**Keywords:** freeskiing, skiing, strength testing, unilateral, isometric, maximal, voluntary, contraction

## Abstract

The aim of the study was to (1) assess the within-session reliability of a unilateral isometric hex bar pull (UIHBP) maximal voluntary contraction (MVC) test and, (2) determine unilateral isometric absolute peak force (PF_abs_) and relative peak force (PF) values in freeski athletes. Twenty-one male and eight female academy to national team freeskiers performed the novel UIHBP MVC task on a force plate and PF_abs_ and relative PF were assessed (1000 Hz). Within-session measures of PF_abs_ offered high reliability on left and right limbs for males (*ICC* = 0.91–0.94, *CV* = 2.6–2.2%) and females (*ICC* = 0.94–0.94, *CV* = 1.4–1.6%), while relative PF measures showed good to high reliability in both left and right limbs for males (*ICC* = 0.8–0.84, *CV* = 2.6–2.2%) and females (*ICC* = 0.92–0.90, *CV* = 1.4–1.7%). We observed significantly lower PF_abs_ (*p* < 0.001) and relative PF (*p* < 0.001) in females compared to males. No statistical difference was found between left and right limbs in males and females in PF_abs_ (*p* = 0.98) and relative PF measures (*p* = 0.93). The UIHBP MVC test appears to be a reliable method for assessing PF_abs_ and relative PF in male and female freeski athletes.

## Introduction

Freeskiing is an extremely complex skill-based action sport that involves numerous technical, tactical, and psychophysical demands (Willmott and Collins, 2015). There are three freeski disciplines (slopestyle, big air and half-pipe). In freeski slopestyle, athletes perform a series of tricks using jumps, custom built rails, and other creative features such as quarter pipes. Freeski big air is performed using only one large jump and competitors perform complex tricks in the air, aiming for high amplitude, style, creative grabs, and a clean landing. During freeski half-pipe, 6–8 tricks are performed whilst skiing down a u-shaped pipe. Accordingly, there are many psychological, skill acquisition and physical factors that may influence the performance, skill execution and safety in freeskiing. To date there is no evidence-based consensus on reliable and practically meaningful physical testing protocols that could be used for screening and monitoring freeski athletes in the context of performance enhancement, injury prevention and/or rehabilitation.

Generally, periodic testing and monitoring of an athlete's neuromuscular performance at several stages during the year can be considered an effective way to provide useful information to practitioners concerning an athletes' current training state (Edwards et al., 2018). This data can be combined with an appreciation and understanding of the emotional load action sport athletes experience (Collins et al., 2018). There is, however, a paucity of data regarding what physical qualities are considered important for freeski athletes from both a supporting performance and injury risk mitigation standpoint. Nevertheless, in certain contexts, maximal strength is plausibly an important capacity to develop for potentially preventing acute and overuse injuries (Lauersen et al., 2018) and is, in certain athletic settings, known to be moderately associated with jump and sprint performance (Kirkpatrick and Comfort, 2013; Comfort et al., 2014). Furthermore, possessing greater lower body strength has been deemed advantageous in other snow sports such as snowboard cross and alpine snowboarding (Vernillo et al., 2016) and alpine skiing (Cross et al., 2021). Nonetheless, a degree of caution should be given when drawing the same conclusions to freeskiing without proper investigation of kinetics, kinematics, and individuals factors such as riding style.

With respect to testing methods, there are numerous approaches to assess athletes' maximal force capabilities. For example, the gold standard method to assess knee flexor and extensor strength is with a motor-driven isokinetic dynamometer (Knapik et al., 1983; Ly and Handelsman, 2002). Isokinetic dynamometry is recommended as it can elicit maximal efforts over a full range of motion (Caruso et al., 2012) and can be used to assess neuromuscular function through different parameters such as peak torque, total work or the peak torque ratio between agonist and antagonist muscles (Gleeson and Mercer, 1996; Bosquet et al., 2016). However, this method is expensive, time consuming and is often impractical in many instances for freeskiers, especially in-season and when testing a group.

Alternative methods to reliably assess maximal strength, are the one-repetition maximum (1 RM) test (Grgic et al., 2020) or via isometric maximal voluntary contraction (MVC) testing at specific joint angles (Drake et al., 2017). In the 1 RM test, eccentric muscles actions are often coupled with concentric actions which can be more reflective of dynamic muscle actions that occur in resistance training and sporting actions (Grgic et al., 2020). Contrasting with isokinetic dynamometry, the 1 RM test is highly cost effective, however this form of testing can also be time consuming with groups of athletes and is also often not appropriate in-season for freeskiers. During isometric contractions, the muscle-tendon unit remains at a constant length and can produce more force than a concentric muscle contraction (Abbott and Wilkie, 1953). Isometric contractions have also been shown to result in reduced structural muscle damage compared to eccentric contractions (Nosaka et al., 2003) which makes this approach of assessment popular in applied settings with athletes. However, isometric contractions performed at longer muscle lengths and for sustained durations can increase muscle soreness, damage, and fatigue (Allen et al., 2018). Finally, common isometric MVC modalities include the isometric leg press (Granacher et al., 2011; Bogdanis et al., 2019), isometric knee extension (Kubo et al., 2006; Noorkõiv et al., 2014), isometric squat (Markovic and Jaric, 2004; Eliassen et al., 2018) and isometric mid-thigh pull (West et al., 2011). These methods are often utilized in training interventions investigating neuromuscular responses to exercise (Taipale et al., 2014), exploring mechanisms of fatigue (Izquierdo et al., 2009) and can also be incorporated into rehabilitation processes (Maestroni et al., 2019; Jordan et al., 2020; Taberner et al., 2020). Despite the advantages of the isometric tests listed above, there are limitations to these methods. For example, these tests often require custom built and robust equipment fixed in place in a laboratory which can create challenges for athletes who travel extensively or train in several locations.

Although the demands of freeskiing have not been quantified, when taking into consideration the incidence and location of injuries often occurring to the knee and lower extremities (Flørenes et al., 2010; Steffen et al., 2017; Palmer et al., 2021), assessing unilateral lower body strength could be warranted. Evaluating athletes' force producing capabilities at several stages during the rehabilitation process can help identify and resolve deficits in neuromuscular performance (Maestroni et al., 2019; Taberner et al., 2020). Moreover, monitoring lower limb strength can provide objective information to help guide task progressions and support inter-disciplinary decision making on important functional milestones such as initiating running, jumping and plyometric activity (Palmieri-Smith and Lepley, 2015; Buckthorpe et al., 2020). The hex bar deadlift also referred to as a ‘trap bar' has become a popular resistance training exercise to perform and is a variant of the barbell deadlift (Camara et al., 2016; Lake et al., 2017; Andersen et al., 2018). Despite this increased popularity, to the authors' knowledge the hex bar deadlift and unilateral variations have not been utilized in testing via the use of force-platforms. Using a hex bar to assess unilateral isometric MVC could provide practitioners with as an alternative testing method when other methods are not compatible or suit their setting and context. However, before using such methods in a practical setting, it is necessary to determine the level of reliability of a test (McCall et al., 2015).

Based on these considerations, the aims of the present study were to (1) evaluate the within-session reliability of absolute (PF_abs_) and relative peak force (PF) during a novel unilateral isometric hex bar pull (UIHBP) maximal voluntary contraction (MVC) test in male and female academy to national team freeski athletes and (2) to provide sex- and level specific reference values.

## Materials and Methods

### Subjects

Twenty-nine academy to national team freeski athletes gave their informed consent to participate in the study: twenty-one males aged 20 ± 2.5 years old, 176 cm ± 3.9, 70 kg ± 3.8, eight females aged 21 ± 4.6 years old, 165 cm ± 2.6, 60.3 kg ± 4.6. Only athletes without a history of knee injuries were included in the study. All participants had to have been enrolled in a freeski academy or part of a national team program. Additional eligibility criteria for the study included having had experience of at least six months of organized strength and conditioning training history and being familiarized with the testing procedures. Subjects did not take part in any physical activity in the 48 h prior to testing. The study was approved by the Ethical Committee of the University of Jyväskylä, and it was conducted according to the provisions of the Declaration of Helsinki.

### Testing Procedures

Testing was conducted to assess the within-session reliability of a UIHBP MVC task. The duration of testing for each subject was 30–45 min. Subjects undertook a 15 min dynamic warm up, consisting of: 5 min of jogging and skipping, 2 min of dynamic stretching, three sets of 6–8 repetitions of bilateral and unilateral ankle pogo jumps, 2 sets of 8 linear and lateral hop and holds, 1 set of 4 squat jumps, 1 set of 4 bilateral and unilateral countermovement jumps and 3 progressive accelerations of 15–20 meters separated by 1 min rest between each acceleration. Three min of passive rest was provided after the completion of the warmup prior to starting the MVC measurements.

### UIHBP MVC Test

**Figure 1:**
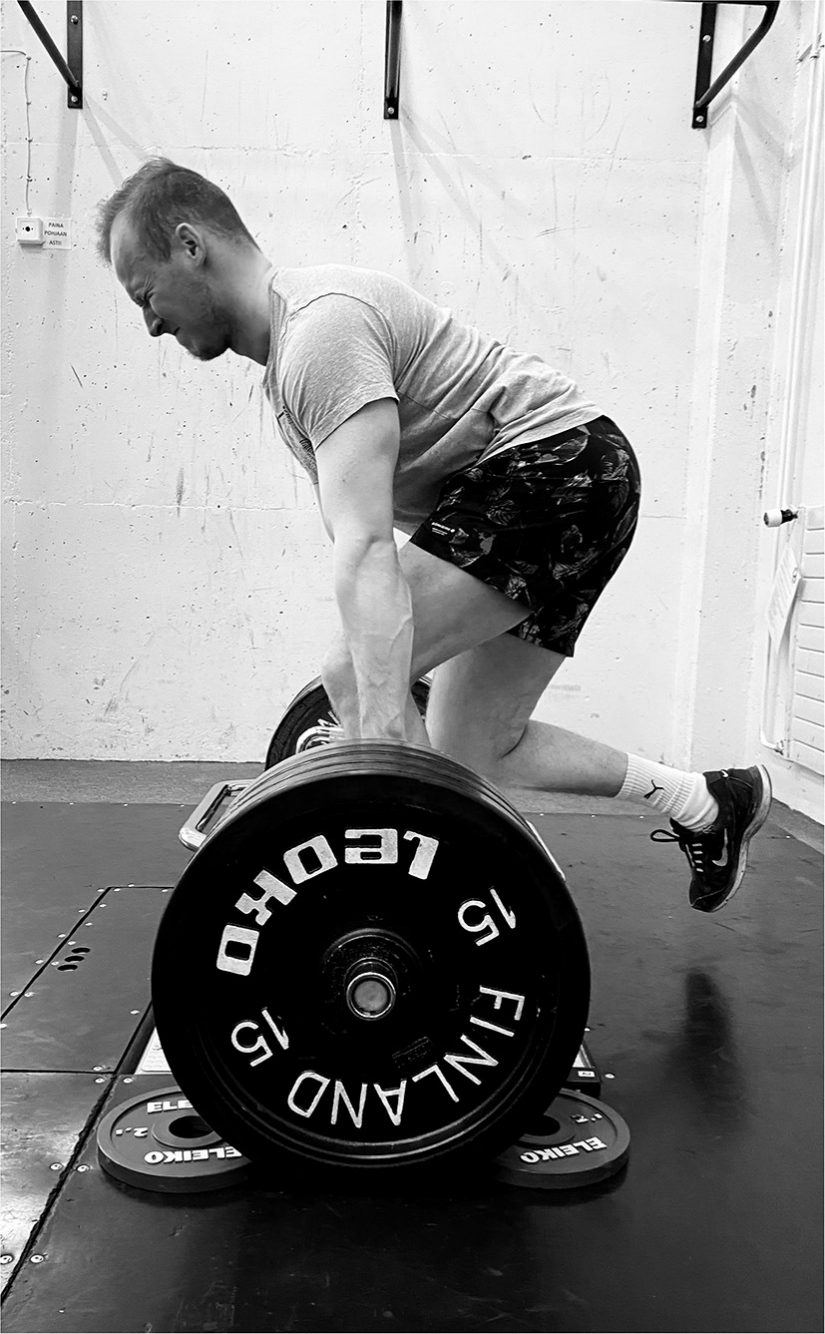
Unilateral maximal isometric hex bar MVC task.

A hex bar (27.5 kg) was loaded with 160 kg to ensure it was secured to the ground and would not move when the subjects were performing the test. The height of the hex bar handle when loaded was 335 mm, the circumference of the handle of the bar was 98 mm. The distance width between the two handles was 588 mm. The subjects were told to prepare as if they were performing a bilateral hex bar deadlift from the ground and a handheld goniometer was used to ensure a knee angle of 115° flexion. If required for taller subjects, blocks were placed under the weights to raise the bar and ensure the correct knee angle was maintained. Once subjects were in the correct starting position and were comfortable, they lifted the uninvolved non-weight bearing limb backwards, ensuring their trunk was in the same position throughout and remained still for 2–3 s before completing warm up trials at 70%, 80% and 90% of self-estimated maximal effort (Figure 1). A second researcher observed the test being performed and was responsible for confirming whether the participant maintained the previously described position during the test. The test was performed following 3 min of rest after the warmup was complete. The subjects were instructed to “pull against the bar and push into the ground as hard as possible” exerting maximal force for 4 s. Each limb was tested 3 times for a total of 3 trials per leg with 60 s rest between each trial. Testing was performed in a training facility and conducted using a force plate (1000 Hz, HUR labs, Finland). The force plate was calibrated before each independent test. PF_abs_ was defined as the maximum force generated during the test and relative PF as PF_abs_ divided by body mass (kg). Coachtech online measurement and feedback system (University of Jyväskylä, Finland) was used to collect the force data (Ohtonen et al., 2016) and was classified according to left and right legs due to the equilateral nature of skiing.

### Statistical Analysis

We confirmed data normality using the Shapiro-Wilk test (Ghasemi and Zahediasl, 2012) and determined within-session reliability (Atkinson and Nevill, 1998) using two-way mixed-effects model ICC with a 95% confidence interval (CI), based on a single measurement (Koo and Li, 2016). Intra-individual coefficient of variation (CV), with a 95% CI, calculated as the average of the CV for each individual where MSE represents the mean squared error across trials and represents the mean of all the trials ($CV=\frac{\sqrt{MSE}\;}{\bar{x}}\times 100\bar{x}$) (Knutson et al., 1994). Reliability thresholds for ICC values were defined as poor (<0.50), moderate (0.50–0.75), good (0.75–0.90), and excellent (>0.90) (Koo and Li, 2016). For coefficient of variation (CV), a value of ≤ 10% was defined as reliable (Brughelli and Van Leemputte, 2013). Sex and leg differences were analyzed using a mixed model two-way analysis of variance. Bonferroni *post-hoc* tests (pairwise comparisons) were performed if significant interactions between group and time were found (VanderWeele and Mathur, 2019). All statistical analyses were conducted with custom-made scripts in MATLAB (Version R2018a, MathWorks, Natick, MA, USA), and statistical significance was set to *p* < 0.05 and confidence intervals to 95%.

## Results

**Figure 2:**
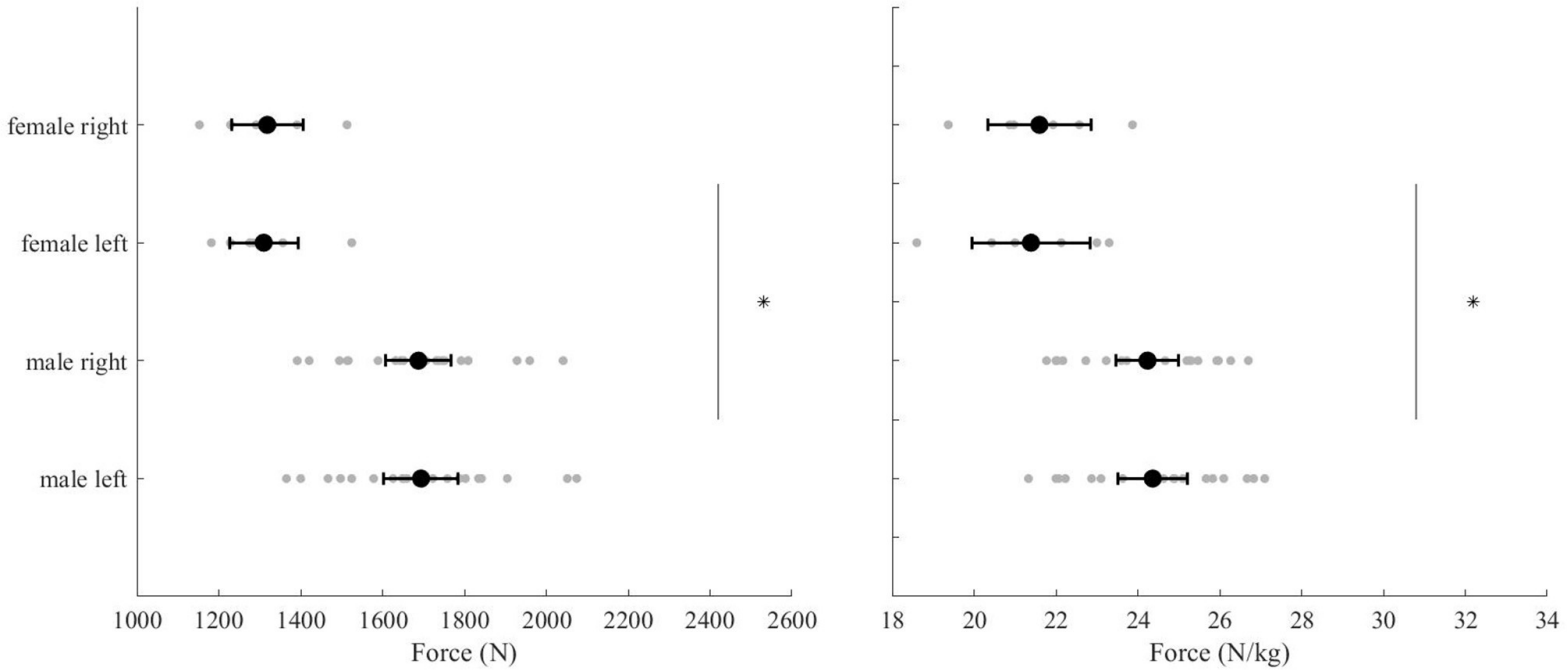
PF_abs_ values (left) for left and right limbs in male and female freeski athletes. Relative PF values (right) for left and right limbs for male and female freeski athletes. Data are shown as mean (+95% confidence intervals) and individual data (gray dots). *Represents a significant difference between male and females.

Within-session reliability variables (ICC, CV, SEM, MDC) and descriptive statistics of male and female PF_abs_ and relative PF values are presented in Tables 1, 2. Within-session measures of PF_abs_ offered high reliability on left and right limbs for males (*ICC* = 0.91–0.94, *CV* = 2.6–2.2%) and females (*ICC* = 0.94–0.94, *CV* = 1.4–2.2%). Relative PF measures showed good to high reliability in both left and right limbs for males (*ICC* = 0.8–0.84, *CV* = 2.6–2.2%) and females (*ICC* = 0.92–0.90, *CV* = 1.4–1.7%). No significant differences in maximal isometric force were observed between left and right legs in either PF_abs_ (*p* = 0.98) or relative PF (*p* = 0.93) measures (Figure 2). Significantly lower maximal isometric force was observed in females compared to males both in PF_abs_ (mean difference (95%CI) = −376 N (−279 to −473), *p* < 0.001) and relative PF (mean difference (95%CI) = −3 N/kg (−2 to −4), *p* < 0.001) (Figure 2). No statistical difference was found between left and right limbs in males and females in PF_abs_ (*p* = 0.98) and relative PF measures (*p* = 0.93).

**Table 1:** Within-session reliability measures of unilateral hex bar isometric pull test.

**UIHBP**	**ICC PF_**ABS**_**	**ICC Relative PF**	**CV% PF_**ABS**_**	**CV%Relative PF**	**SEM (N)**	**MDC (N)**
Male left	0.91 (0.80–0.96)	0.8 (0.55–0.91)	2.6% (1.9–3.3)	2.6% (1.8–3.3)	59	162
Male right	0.94 (0.87–0.98)	0.84 (0.62–0.93)	2.2% (1.8–2.7)	2.2% (1.7–2.7)	44	122
Female left	0.94 (0.74–0.99)	0.92 (0.66–0.98)	1.4% (0.8–2)	1.4% (0.78–2.2)	21	68
Female right	0.94 (0.87–0.97)	0.9 (0.55–0.98)	1.6% (0.6–2.5)	1.7% (0.6–2.8)	27	75

*ICC, intraclass correlation coefficient; CV, coefficient of variation; PF_abs_, absolute peak force; PF, peak force; SEM, standard error measurement; MDC, minimal detectable change*.

**Table 2:** Descriptive statistics for unilateral isometric hex bar pull outcome variables.

**UIHBP**	**Mean PF_**ABS**_ (N)**	**Mean relative PF (N/kg)**
Male left	1708.7 ± 183	24.6 ± 1.7
Male right	1697.6 ± 195	24.5 ± 1.7
Female left	1318.9 ± 97	21.5 ± 1.3
Female right	1339.5 ± 106	21.9 ± 1.02

*PF_abs_, absolute peak force; PF, peak force. Data are shown as mean ± 1 standard deviation*.

## Discussion

The main finding of this study was that the UIHBP MVC test when performed on a force plate offered good-excellent within-session reliability in PF_abs_ and relative PF in both male and female freeski athletes. This study also provided force production data of the lower body in male and female freeski athletes. It was found that female freeski athletes produced lower PF_abs_ and relative PF when compared to male freeskiers.

Reliable testing methods and protocols are required to confidently detect meaningful changes in performance (Moeskops et al., 2018). The findings from this study offer practitioners a viable option to assess unilateral lower body strength. ICC values between 0.75 and 0.9 indicate good reliability, and values <0.90 indicate excellent reliability (Portney and Watkins, 2009; Koo and Li, 2016). The ICC calculated for the male left and right limbs for PF_abs_ were 0.91–0.94 and for relative PF were 0.8–0-84. The ICC for the female left and right limbs for PF_abs_ was 0.94–0.94 and for relative PF was 0.92–0.90. The CV is a common and robust criterion to test reliability and a CV of ≤ 10% is often used as the criterion to declare a variable as reliable (Brughelli and Van Leemputte, 2013). The CV calculated for the male left and right limbs for PF_abs_ were 2.6%–2.2% and for relative PF were 2.6–2.2%. The CV for female left and right limbs for PF_abs_ were 1.4%-1.6% and for relative PF were 1.4–1.7%. A force plate is the gold standard for measuring isometric muscle force (Verdera et al., 1999). Therefore, providing practitioners follow the same testing procedures as presented in the current study, they can be confident that they are collecting reliable data from their athletes. The UIHBP MVC test appears to offer equivalent reliability values when compared to the isometric mid-thigh pull and the isometric squat (*ICC* = ≥ 0.80 to 0.99) (Drake et al., 2017) and therefore, offers an alternative method for assessing unilateral PF when other methods are not appropriate or feasible.

This is the first study to present data regarding unilateral lower body strength values of male and female freeskiers. As expected, significantly lower maximal isometric force in females compared to males in both PF_abs_ (*p* < 0.001) and relative PF capacities (*p* < 0.001) were observed. The main factors accounting for differences in lower body strength between men and women are likely due to muscle mass (Miller et al., 1993), greater proportion of fast type fibers (Nindl et al., 1995) and morphological characteristics such as muscle thickness, pennation angle, and fascicle length (Blazevich and Sharp, 2005; Bartolomei et al., 2019). Furthermore, although knee flexion was controlled for in this study, hip flexion was not, and this may have contributed to a certain extent to the differences between sexes. There was no statistical difference between left and right limb absolute PF_abs_ (*p* = 0.98) or relative PF (*p* = 0.93) measures. Freeskiing is an equilateral sport characterized by similar physical demands on each leg. However, certain tricks and the initiation of aerial maneuvers and landings often occur using predominantly one leg. Consequently, freeskiers may commonly utilize various forms of unilateral resistance training to seek a desired training adaptation. These findings could therefore be of potential interest for practitioners working with freeski populations. However, unilateral strength measurement values recorded in the laboratory may not be correlated to the ground reaction force kinetics in the sport (Ogrin et al., 2021). Further research is required to determine a detailed physiological and neuromuscular profile of academy to elite freeski athletes. Such data combined with kinetic, kinematic, and qualitative analysis of tricks during the sport could provide meaningful information to practitioners aiming to enhance the physical preparation of these athletes and help support talent identification and long-term athlete development models. Without further investigation, it is uncertain whether the UIHBP test could be used to track both acute and chronic changes in neuromuscular performance. Nevertheless, isometric contractions have been shown to be a highly reliable means of assessing and tracking force production (Wilson and Murphy, 1996; Bazyler et al., 2015; Drake et al., 2017). However, the ability of isometric assessments to predict dynamic performance compared to alternative modalities of assessment such as isokinetic and isoinertial testing is not as well supported (Wilson and Murphy, 1996).

Recent data from the 2016 and 2020 Youth Winter Olympic Games show that the highest percentage of injuries occurred in freeski and snowboard slopestyle disciplines (Steffen et al., 2017; Palmer et al., 2021). A similar trend was also apparent at the PyeongChang 2018 Winter Olympic Games (Soligard et al., 2019). This highlights that further longitudinal/multi-season injury surveillance data of academy to elite level freeskiers and in-depth investigation of injury mechanisms are required. Previous data from Flørenes et al. (2010) showed that one quarter of all injuries encountered by freestyle ski athletes involved the knee (with 38% of these relating to the ACL). However, this study included the freestyle ski disciplines of moguls, dual-moguls, aerials, ski cross and halfpipe skiers, with no data from the freeski disciplines of slopestyle and big air. Given that it is common for freeski athletes to suffer an injury, it would appear worthwhile to monitor training load and neuromuscular status throughout the season and during appropriate stages of the return to sport process. The UIHBP MVC test outlined in the present study could potentially be incorporated into lower body rehabilitation programs. Physical properties such as maximum strength, explosive strength, and reactive strength have also been shown to influence reinjury outcomes (Kyritsis et al., 2016; King et al., 2018) and it is recommended that objective physical testing be carried out before athletes return to sport (Carolan et al., 2020). Regarding maximal strength, there is evidence highlighting that return to sport frameworks should include the assessment of unilateral quadriceps MVC during suitable phases of the rehabilitation (Buckthorpe et al., 2020; Jordan et al., 2020). Current ACL return to sport protocols recommend using a quadriceps limb symmetry index of 90% before determining readiness to return to sport (Gokeler et al., 2016; Brown et al., 2021), though it is not clear whether the same thresholds are appropriate for freeski athletes. It is important to note that although objective insight into elements of the recovery process can be useful, isometric quadriceps limb symmetries can overestimate the recovery of the injured limb (Wellsandt et al., 2017) and therefore, consideration must be given when examining the data of such measures in isolation. Practitioners are recommended to consider a holistic, multifactorial and individual approach to injury rehabilitation (Lahti et al., 2020), emphasizing movement quality (Buckthorpe, 2021), tasks that incorporate decision making and divided attention (Hughes and Dai, 2021), as well as nutritional (Shaw et al., 2019) and psychological readiness elements (Papadopoulos et al., 2018; D'Astous et al., 2020).

It must be highlighted that this data is only representative of the current cohort of freeski athletes recruited in this study and further data is required, especially from female athletes as the sample size in this study was small. Additionally, these measurements and comparisons were only taken from one specific joint angle (115° knee flexion). Further analysis from several knee and hip angles could yield different results as alternative limb arrangements can affect force production and muscle recruitment patterns (Dos' Santos et al., 2017; Goodwin and Bull, 2021). However, isometric contractions performed at longer muscle lengths can result in increased muscle damage (Allen et al., 2018). Moreover, correlation with an isokinetic dynamometer and further exploration of the current test via kinematic and EMG analysis to accurately quantify and compare muscle activation patterns could help practitioners make a more informed decision regarding test selection for their environment and specific needs. A limitation to the study is that between-session reliability was not assessed. However, it was not possible to do so without interfering with the day-to-day training of the athletes. Furthermore, rate of force development (RFD) was not analyzed in the current study. Attempting to achieve maximal force and RFD within the same contraction may result in suboptimal measures of both parameters (Maffiuletti et al., 2016). It is recommended that when assessing RFD that contractions be “fast and hard” with short durations (0.5–1.5 s) (Maffiuletti et al., 2016) and this would have interfered with the specific aim of the current study to establish the reliability of measuring peak force.

## Conclusion

The UIHBP MVC test can be considered as a simple and quick testing approach that provides reliable measures of lower body PF_abs_ and relative PF. The study also provided unilateral lower body force production reference values for male and female freeski athletes.

## Data Availability Statement

The raw data supporting the conclusions of this article will be made available by the authors, without undue reservation.

## Ethics Statement

The studies involving human participants were reviewed and approved by University of Jyväskylä ethics committee. Written informed consent to participate in this study was provided by the participants' legal guardian/next of kin. Written informed consent was obtained from the individual(s) for the publication of any potentially identifiable images or data included in this article.

## Author Contributions

JM designed the study and concept under the supervision of VL and JS. JM conducted all data collection and measurements. BG performed the statistical analysis. JM wrote the first draft. VL, JS, and BG significantly contributed to the final version. All authors contributed to the article and approved the submitted version.

## Conflict of Interest

The authors declare that the research was conducted in the absence of any commercial or financial relationships that could be construed as a potential conflict of interest.

## Publisher's Note

All claims expressed in this article are solely those of the authors and do not necessarily represent those of their affiliated organizations, or those of the publisher, the editors and the reviewers. Any product that may be evaluated in this article, or claim that may be made by its manufacturer, is not guaranteed or endorsed by the publisher.
